# Development and Validation of a Cross-Cultural Knowledge, Attitudes, and Practices Survey Instrument for Chronic Kidney Disease in a Swahili-Speaking Population

**DOI:** 10.1371/journal.pone.0121722

**Published:** 2015-03-26

**Authors:** John W. Stanifer, Francis Karia, Corrine I. Voils, Elizabeth L. Turner, Venance Maro, Dionis Shimbi, Humphrey Kilawe, Matayo Lazaro, Uptal D. Patel

**Affiliations:** 1 Department of Medicine, Duke University, Durham, North Carolina, United States of America; 2 Duke Global Health Institute, Duke University, Durham, North Carolina, United States of America; 3 Kilimanjaro Christian Medical College, Moshi, Tanzania; 4 Health Services Research and Development, Durham Veterans Affairs Medical Center, Durham, North Carolina, United States of America; 5 Department of Biostatistics and Bioinformatics, Duke University, Durham, North Carolina, United States of America; 6 Duke Clinical Research Institute, Duke University, Durham, North Carolina, United States of America; Rajarata Univeresity of Sri Lanka, SRI LANKA

## Abstract

**Introduction:**

Non-communicable diseases are a growing global burden, and structured surveys can identify critical gaps to address this epidemic. In sub-Saharan Africa, there are very few well-tested survey instruments measuring population attributes related to non-communicable diseases. To meet this need, we have developed and validated the first instrument evaluating knowledge, attitudes and practices pertaining to chronic kidney disease in a Swahili-speaking population.

**Methods and Results:**

Between December 2013 and June 2014, we conducted a four-stage, mixed-methods study among adults from the general population of northern Tanzania. In stage 1, the survey instrument was constructed in English by a group of cross-cultural experts from multiple disciplines and through content analysis of focus group discussions to ensure local significance. Following translation, in stage 2, we piloted the survey through cognitive and structured interviews, and in stage 3, in order to obtain initial evidence of reliability and construct validity, we recruited and then administered the instrument to a random sample of 606 adults. In stage 4, we conducted analyses to establish test-retest reliability and known-groups validity which was informed by thematic analysis of the qualitative data in stages 1 and 2. The final version consisted of 25 items divided into three conceptual domains: knowledge, attitudes and practices. Each item demonstrated excellent test-retest reliability with established content and construct validity.

**Conclusions:**

We have developed a reliable and valid cross-cultural survey instrument designed to measure knowledge, attitudes and practices of chronic kidney disease in a Swahili-speaking population of Northern Tanzania. This instrument may be valuable for addressing gaps in non-communicable diseases care by understanding preferences regarding healthcare, formulating educational initiatives, and directing development of chronic disease management programs that incorporate chronic kidney disease across sub-Saharan Africa.

## Introduction

Non-communicable diseases (NCDs) disproportionately affect the economic, social and health outcomes of low- and middle-income countries. Understanding knowledge, attitudes and practices associated with NCDs is vital to addressing the rising global epidemic [1]. Cross-cultural surveys that measure these attributes are valuable; however, ethnic, social, historical and language differences that often exist between the researchers and those study populations mean that many surveys are not appropriate or easily adaptable for use across various settings of interest [2].

Chronic kidney disease (CKD) is an NCD with high cardiovascular morbidity and mortality, and management programs have been shown to reduce the incidence of end-stage kidney disease in low-income regions where it is a fatal condition [3]. The Comprehensive Kidney Disease Assessment for Risk Factors, epIdemiology, Knowledge, and Attitudes (CKD-AFRIKA) study is an ongoing project in northern Tanzania with the aim of understanding the epidemiology, etiology, knowledge, attitudes and practices associated with CKD as well as other related NCDs. As part of our work, we have developed the first knowledge, attitudes and practices (KAP) survey pertaining to issues of CKD and chronic disease among a Swahili-speaking population. In Tanzania there are 35 million native speakers of Swahili, and it is the *lingua franca* of East Africa where more than 120 million people across eight countries speak it making it the most widely spoken language in Africa [4–6].

We present here a mixed-methods approach that we used in order to develop, validate and refine our cross-cultural survey instrument. We present the results of the validation process for our KAP survey instrument as well as the tool itself so that other researchers may use it in similar populations. We also demonstrate the iterative process that is required to create a cross-cultural survey and provide enough detail and transparency to make these methods accessible to researchers in multiple disciplines. Our methods can be used as a guide for those conducting related research in NCDs.

## Methods and Results

### Ethics Statement

The study protocol was approved by Duke University Institutional Review Board (#Pro00040784), the Kilimanjaro Christian Medical College (KCMC) Ethics Committee (EC#502) and the National Institute of Medical Research (NIMR) in Tanzania. Additionally, we obtained approval from each local district or neighborhood leader prior to any recruitment from their respective jurisdiction. Written informed consent was obtained from all participants.

### Study Setting

The study was conducted between December 2013 and June 2014 in the Kilimanjaro Region of Tanzania. This region has an adult population of more than 900,000 people. Almost 35% of the population lives in urban areas, and it is nearly evenly divided between men and women [7]. Currently more than 95% of school-age children are enrolled in primary school, and literacy rates are greater than 80% for both men and women which place the region above the national average. The largest ethnic group is the Chagga tribe followed by the Pare, but the Maasai and Sambaa tribes also have a presence. In total, over 120 ethnic groups reside in Tanzania, and Swahili is the major language of the region and the entire country [8]. All participants in our study spoke Swahili as their first language.

### Development of the Survey Tool

**Fig 1:**
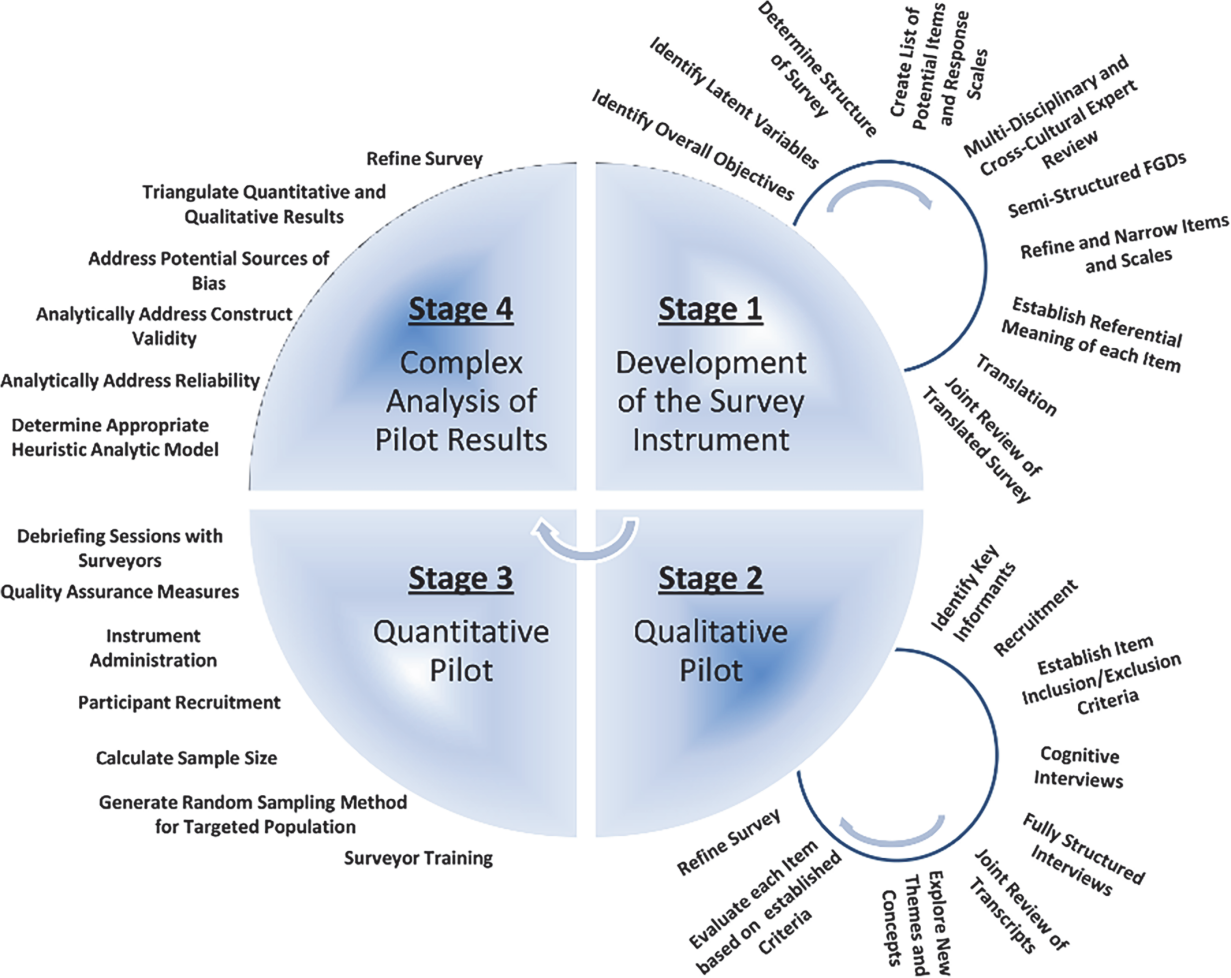
Action Cycle Outlining the Iterative Process for Developing and Piloting the Cross-Cultural Survey Instrument.

The process used to develop our survey was composed of four major stages each consisting of several steps (**Fig. 1**). This systematic approach enabled a qualitative and quantitative iterative assessment of the survey tool and emphasized the dynamic nature of survey development. Reporting of our qualitative methods meets the Consolidated Criteria for Reporting Qualitative Research (COREQ) checklist [9], and reporting of our mixed-methods protocol meets the Mixed Methods Appraisal Tool (MMAT) Criteria [10]. Here we describe the four stages in detail including both methods and results for each stage.

### Stage 1. Development of the Survey Instrument

#### Methods

Prior to the development of this survey instrument, we performed a systematic literature review in order to shape our objectives [11]. We identified key knowledge gaps that must be bridged in order to effectively formulate treatment and prevention programs for NCDs that incorporate CKD. One such gap is the limited understanding of peoples’ knowledge, attitudes and practices associated with kidney disease. Therefore, we developed a structured instrument comprising three domains each designed to test these attributes in a Swahili-speaking population (**S1 Appendix**).

In the first domain, items were constructed to test knowledge of the etiology, diagnosis, symptoms and outcomes of kidney disease and were measured on a four-point categorical response scale (‘Yes’, ‘No’, ‘Do Not Know’ and ‘Unsure’). In the second domain, items were constructed to test attitudes related to kidney disease, and they were measured on a dichotomous response scale (‘Yes’ and ‘No’). In the third domain, items were constructed to test hypothetical practices associated with a diagnosis of kidney disease, and they were measured on a four-point Likert-based scale (‘Very Unlikely’, ‘Unlikely’, ‘Likely’ and ‘Very Likely’). The relationship between the items and their respective constructs (i.e. knowledge, attitudes or practices) was represented by a casual indicator model because the items collectively determined the construct. Causal indicators are non-redundant items that capture the range of a latent construct, and they are informed by literature reviews, expert opinions and qualitative data collections from members of the target population to capture. The causal indicator model is different from the more commonly used effect indicator model in which the latent construct determines the values of the survey items [12, 13].

Cross-cultural experts from multiple disciplines including medicine, global health, anthropology, sociology and biostatistics generated the items and the corresponding response scales. Consensus was obtained for each item before its inclusion in the survey, and we included a mixture of both truly and falsely worded items designed to test knowledge [14]. After addressing formatting issues, the referential meaning of each survey item was established. This not only clarified our intended meaning of each item (and the latent variable being measured), but it served as a reference for the surveyors during the piloting sessions. It also served as an additional means of addressing the codability of words and concepts as they were translated from English to Swahili. In cross-cultural surveys where local behaviors, patterns and worldviews can be very different, this step was especially important [15].

To ensure that the items covered issues relevant to the local population (content validity), as required for causal indicator models, we conducted four focus group discussions (FGDs) composed of 55 participants in December 2013. The FGDs included adult men and women of all ages from the general population, the hospital medicine clinics and traditional healers’ clinics. We considered these participants to be the key informants as they represented a spectrum of well-adults as well as chronically-ill adults seeking care from both biomedical and traditional clinics. These sessions were semi-structured, and the probe questions were based on the pilot survey items. Each FGD was led by a moderator native to the area (FK), and they were all audio-recorded, transcribed and independently translated by two research assistants (DS and HK) who also served as note takers for the sessions. Debriefings were held after each session, and team meetings were again held following translation. We used these sessions to identify important content that we had not otherwise included in the survey instrument, and as a direct result of the emerging content of these sessions, four items (Items 3, 5, 21, and 22 of the final version of the instrument) were added.

#### Results

Following these sessions, a final pilot version of the survey instrument was constructed in English and was reviewed by the lead investigators (JWS and FK). The instrument was then independently translated into Swahili by two native speakers one of whom is a trained medical professional. After back-translation, we conducted a joint review of each version that included the investigators and translators. Together we explored issues in codability by identifying words and concepts with difficult translations, and we formulated a single pilot version of the instrument.

### Stage 2. Qualitative Pilot

#### Methods

Issues that may negatively impact construct validity in cross-cultural surveys include translation difficulties with individual words or entire concepts (codability), different intended or referential meaning between the researchers and participants, structural issues in the format and order of the survey itself and sensitivity of the questions. To address all of these potential issues, we conducted qualitative piloting sessions. Before piloting, we first adopted an existing set of twelve criteria by which to judge each item in the local context [15, 16]. In order to be included each item had to meet all of the adopted criteria (**Table 1**).

**Table 1:** Inclusion criteria for survey items.

**Criteria**
**1. Level of language (Swahili) was understandable**
**2. The question was simple and grammatically correct**
**3. Singular and plural ‘you’ was clear**
**4. Reflected local health concerns**
**5. Meaning and interpretation was clear**
**6. The question made sense to everyone**
**7. A ‘yes’ was clear and unambiguous in meaning**
**8. A ‘no’ was clear and unambiguous in meaning**
**9. Time period was clear**
**10. The question was not viewed as too sensitive**
**11. Appropriate degree of discrimination (i.e. a range of positive and negative responses)**
**12. Resulted in meaningful answers**

We first piloted the instrument using 16 cognitive interviewing sessions in January 2014 [17]. Using purposive sampling, we again targeted key informants which included adults from the general population, chronically-ill patients from the hospital medicine clinics and patients from traditional healers and herbal vendors. We targeted both men and women of all ages from urban and rural settings with varying education levels and ethnicities. The majority of participants were men (n = 11; 69%), and the age range was 19 to 60 years. Half the participants had only a primary education (n = 8; 50%), and four participants (25%) had a university level education. Six (38%) were Maasai and six (38%) were Sambaa.

All 16 sessions were audio-recorded and then transcribed and translated by two local research assistants (DS and HK). Each session followed a semi-structured protocol. First, the surveyor was instructed to ask the participant(s) what he/she thought was meant by each question. Next, the surveyor read the referential meaning to the participant(s) and proceeded to ask a series of probing questions. The probe questions were designed to assess four key areas of the response process: comprehension, retrieval, judgment and response. At the completion of each session, a debriefing was held which included translators, surveyors, note takers and researchers. We continued this process until no further changes were required to the instrument.

Following these cognitive interviewing sessions, we conducted structured interviews (n = 50) with adults from the general population. These interviews simulated the actual field-administration of the survey instrument. The surveyor read the informed consent, collected demographic data, read the instructions and then verbally administered the entire survey as if it were being done for field data collection. At the end of each session, the participant was asked to complete a series of structured questions pertaining to formatting, ability to follow instructions, the order of the survey questions and the appropriateness of the answer choices.

#### Results

As a result of the cognitive interviews, 12 items were modified: two items underwent significant modification, including the addition of an introductory statement for one; eight items underwent minor modification due to translation or formatting issues; one item was removed due to poor discrimination; and one item was removed due to comprehension and response difficulties. In the end, these interviews suggested that all translated items included in the final pilot version of the survey reflected the underlying referential meanings. Finally, these piloting sessions also indicated that each item, its referential meaning and its response scale corresponded to and measured the latent variables they were designed to test. As a result of observations based o the additional structured interviews, five items underwent minor formatting modifications.

### Stage 3. Quantitative Pilot

#### Methods

After two surveyors (DS and HK) were trained in the administration of the survey, a random sample of participants was obtained using cluster probability sampling. From 230 total administrative neighborhoods, thirty seven were randomly chosen from the Moshi Urban and Moshi Rural districts based on probability proportional to size [7], and within each neighborhood, a cluster site was determined using geographic points randomly generated by Arc Global Information Systems (ArcGIS), v10.2.2 (Environmental Systems Research Institute, Redlands, CA). Households were then randomly chosen based on coin-flip and die-rolling techniques according to our established protocol (**S2 Appendix**). All adults (age ≥ 18 years old) who were Tanzanian citizens were eligible for inclusion.

We targeted a minimum number of 520 participants to ensure a participant-to-item ratio (N/*p*) of at least 20:1 [18]. To reduce non-response rates, we attempted a minimum of two additional visits on subsequent days and weekends. When eligible participants were not at home, we obtained permission and mobile phones numbers from family members to locate them. Additionally, when available, we collected basic demographic data for the non-responders.

We collected demographic information for each participant, and we then administered the survey verbally in Swahili in order to reduce recall bias and provide more culturally-consistent answers (i.e. reduce cultural frame switching) [19]. Both surveyors were instructed to answer questions related to clarifying instructions and words, but they were instructed to not answer questions related to the meaning of each question. When possible, the instrument was administered in private in order to reduce the response bias that the presence of other persons may have had. Both surveyors were instructed to record any abnormalities that occurred during administration such as interruptions or excessive answer-changing. Among a random and feasible number of participants (n = 32), the instrument was administered again under similar circumstances within a one to three week period to establish test-retest reliability.

Throughout this stage, regular debriefing sessions were held between the surveyors (DS and HK) and the lead investigators (JWS and FK). All data were collected on paper and then electronically entered into the study’s purpose-built Research Electronic Data Capture (REDCap) database. After data entry, all data were verified by an independent reviewer to ensure accuracy. To ensure that the sampling protocol was strictly adhered to, the principle investigator (JWS) regularly accompanied the surveyors into the field and routinely conducted anonymous shadowing of the surveyors.

We used STATAv.13 (STATA Corp., College Station, TX) to analyze the demographics and survey instrument results. Continuous variables are reported as median (inter-quartile range [IQR]) and categorical variables as counts and percentages. We compared differences among groups using the chi-square test for independence. All p values are two-sided. We accounted for the design effect due to cluster sampling by using the Taylor Series linearization method which was implemented using the svy suite of commands in STATA.

#### Results

Between January and May of 2014, 606 participants were enrolled from 431 households. The household non-response was 15%. The total sample consisted of 149 men (24.6%) and 457 women (75.4%) with a median age of 43.0 years (IQR 32–57). The majority of participants were Chagga (n = 363; 59.9%), farmers or daily wage-earners (n = 258; 39.4%), educated to the level of primary school (n = 451; 74.4%) and lived in an urban setting (n = 463; 76.4%)(**Table 2**). Participants living in an urban setting were significantly more likely to have a secondary (p = 0.01) or post-secondary (p = 0.02) education, consider themselves unemployed (p<0.001) or work in a self-employed small business/vendor (p<0.001) or in a professional occupation (p = 0.05). They were also slightly younger with fewer participants being over the age of 60 years (p = 0.05).

**Table 2:** Demographic and social characteristics of the survey respondents.

**Category**	**Variable**	**Urban (n = 463)**	**Rural (n = 143)**	**Total Number(n = 606)**	**p-value** ^††^
**Demographics**	**Gender (%)**				
	Male	112 (24.2)	37 (25.9)	149 (24.6)	0.61
	Female	351 (75.8)	106 (74.1)	457 (75.4)	0.61
	**Age (%)**				
	18–39 years old	203 (43.8)	45 (31.5)	248 (40.9)	0.11
	40–59 years old	169 (36.5)	56 (39.1)	225 (37.1)	0.54
	60+ years old	91 (19.7)	42 (29.4)	133 (22.0)	0.05
	**Ethnicity (%)**				
	Chagga	285 (61.6)	78 (54.6)	363 (59.9)	0.64
	Pare	44 (9.5)	35 (24.5)	79 (13.1)	0.42
	Sambaa	22 (4.8)	11 (7.7)	33 (5.5)	0.63
	Other*	112 (24.1)	19 (13.3)	131 (21.5)	0.17
**Socials Characteristics**	**Education (%)**				
	None	25 (5.4)	4 (2.8)	29 (4.8)	0.37
	Primary	325 (70.2)	126 (88.1)	451 (74.4)	<0.001
	Secondary	80 (17.3)	12 (8.4)	92 (15.2)	0.01
	Post-Secondary	33 (7.1)	1 (0.7)	34 (5.6)	0.02
	**Occupation (%)**				
	Unemployed^#^	81 (17.5)	6 (4.2)	136 (20.8)	<0.001
	Farmer/Wage Earner	149 (32.2)	109 (76.2)	258 (39.4)	<0.001
	Small Business/Vendors	179 (38.7)	19 (13.3)	198 (30.2)	<0.001
	Professional^†^	54 (11.6)	9 (6.3)	63 (9.6)	0.05

*Other Tribal Ethnicities represented in our groups include Maasai, Luguru, Kilindi, Kurya, Mziguwa, Mnyisanzu, Rangi, Jita, Nyambo and Kaguru

# Includes housewives and students

† Professional includes any salaried position (e.g. nurse, teacher, government employee, etc.) and retired persons

††For comparison of urban and rural

Among the 32 participants who were administered the instrument twice, 21 (65.6%) were female and the median age was 42.0 years (IQR 36–59). The majority were Chagga (n = 18; 56.3%), small business self-employed (n = 12; 37.5%) and educated to the level of primary school (n = 27; 84.4%).

One item (Item 26), which asked how likely the person would be to tell his/her family if he/she were diagnosed with kidney disease, was removed from the final version of the instrument during this stage. Although the item tested well in the piloting sessions (Stage 2), fewer than 1% of participants responded negatively (very unlikely or unlikely) to this item suggesting it did not discriminate different levels of knowledge.

### Stage 4. Complex Analysis of Pilot Results

#### Methods

Based on *a priori* expectations about the purpose of the survey instrument, we used a causal indicator model for our heuristic analyses. Specifically, all items for each domain of our survey were designed to capture the range of that latent construct so that no item was redundant, and thus consistent with the requirements for a casual indicator model. Without causal indicators, internal consistency may be speciously low because the items represent different components of the same underlying trait (e.g. etiology of kidney disease and consequences of kidney disease) [12]. As such, the items may be weakly correlated (positively or negatively) or uncorrelated [13]. Consequently, causal indicators also are not amenable to factor analysis.

To test the assumption that the data fit a causal indicator model, we examined inter-item correlations in a polychoric correlation matrix which is a better estimator for binary or ordinal scales than the more commonly used Pearson’s matrix for continuous scales [20, 21]. The polychoric correlation matrix confirmed that the survey instrument was best measured using a causal indicator model. Items within the knowledge domain were positively but weakly correlated (average inter-item correlation = 0.29) (**Table 3A**), and the items from the attitudes (average inter-item correlation = 0.20) and practices (average inter-item correlation = 0.27) domains showed weak positive and negative correlations (**Table 3B, C**).

**Table 3:** a-c, Polychoric correlation matrices for the three domains of knowledge, attitudes, and practices based on results of the 606 adults who completed the piloted survey instrument.

**3a. Correlation among items within the knowledge domain** *										
	Item 1	Item 2	Item 3	Item 4	Item 5	Item 6	Item 7	Item 8	Item 9	Item 10
Item 1 (blood pressure…?)	1.00									
Item 2 (diabetes…?)	0.58	1.00								
Item 3 (alcohol…?)	0.29	0.34	1.00							
Item 4 (a person can tell…?)	0.35	0.28	0.33	1.00						
Item 5 (diagnosis…?)	0.13	0.22	0.28	0.49	1.00					
Item 6 (prevention…?)	0.10	0.07	0.12	0.28	0.41	1.00				
Item 7 (temperature…?)	0.30	0.28	0.25	0.30	0.21	0.20	1.00			
Item 8 (filter waste…?)	0.15	0.23	0.32	0.30	0.36	0.34	0.38	1.00		
Item 9 (dialysis…?)	0.22	0.27	0.16	0.30	0.30	0.27	0.25	0.33	1.00	
Item 10 (antibiotics…?)	0.17	0.12	0.25	0.22	0.25	0.06	0.34	0.28	0.04	1.00
**3b. Correlation among items within the attitudes domain** †										
	Item 11	Item 12	Item 13	Item 14	Item 15	Item 16	Item 17	Item 18		
Item 11 (thought about…?)	1.00									
Item 12 (learning about…?)	-0.13	1.00								
Item 13 (worried about…?)	0.10	-0.17	1.00							
Item 14 (reputation…?)	-0.05	0.05	0.60	1.00						
Item 15 (ability to work…?)	-0.07	0.06	0.64	0.81	1.00					
Item 16 (survival…?)	0.11	0.02	0.43	0.61	0.64	1.00				
Item 17 (community problem…?)	0.11	-0.22	0.20	0.15	0.20	0.12	1.00			
Item 18 (cost of…?)	0.16	-0.19	0.23	0.27	0.30	0.26	0.28	1.00		
**3c. Correlation among items within the practices domain** ‡										
	Item 19	Item 20	Item 21	Item 22	Item 23	Item 24	Item 25	Item 26		
Item 19 (…from a healer?)	1.00									
Item 20 (…at home?)	0.54	1.00								
Item 21 (…hospital?)	-0.02	0.06	1.00							
Item 22 (…cell phone?)	0.00	0.00	0.46	1.00						
Item 23 (…email?)	0.16	0.18	-0.05	0.13	1.00					
Item 24 (use of herbals…?)	0.65	0.50	0.19	0.03	0.11	1.00				
Item 25 (see Medical Doctor…?	0.02	0.02	0.40	0.38	-0.04	0.09	1.00			
Item 26^††^ (tell family…?)	0.57	0.57	0.54	0.55	0.47	0.59	0.52	1.00		

*The response scale consisted of *Yes*, *No*, *Do not know* and *Unsure* with *Do not know* and *Unsure* coded as one response.

†The response scale consisted of *Yes* and *No*

‡The response scale consisted of *Very Unlikely*, *Unlikely*, *Likely* and *Very Likely*

††Item 26 was removed from the final version of the instrument

In causal indicator-based scales, total scores may be derived from any number of combinations of responses to the individual items. Therefore, the test-retest reliability was evaluated for each item rather than the total scale by estimating the intra-cluster correlation (ρ) as well as Spearman’s rank correlation coefficient.

Construct validity of the knowledge domain was tested using the known-groups method [22, 23]. To establish divergence among groups we used the Framework Method to perform a thematic analysis of the qualitative data from Stages 1 and 2 [24]. The coding and corresponding matrices were stored and analyzed using NViVOv10.0 (QRS International Pty Ltd, Melbourne, Australia). This method allowed us to systematically develop expectations for convergent and divergent survey responses among different social groups (e.g. urban and rural populations). Differences in means between the known groups who completed the survey instrument in Stage 3 were calculated using a Student’s T test or Analyses of Variances (ANOVA).

#### Results

For each item, the intra-class correlation (ρ) was > 0.70 and Spearman’s coefficient was >0.70 with a p value < 0.05 indicating that the survey demonstrated excellent test-retest reliability among the 32 participants who completed the survey twice. With regard to known-groups validity, results of the Framework Method indicated that younger participants and educated participants should be expected to score higher on the knowledge domain of the instrument. Because urban participants (p = 0.001), men (p<0.001), and professional (p<0.001) were significantly more likely to have an education beyond the primary level, we also expected that these groups would score higher on the knowledge domain.

In order to test for the presence of these expected differences among groups who completed the survey instrument in Stage 3, we calculated a summary score for the knowledge domain and compared the means. We scored each as correct (1) or incorrect (0) with the responses ‘Do Not Know’ and ‘Unsure’ treated as incorrect.

We observed the expected differences in mean scores (**Table 4**). As expected, men (p<0.001), professionals (p = 0.06) and urban participants (p = 0.002) scored higher. Unexpectedly, unemployed participants also scored higher (p = 0.04); however, twelve of these participants (14%) had a post-secondary education and the mean score dropped to 3.60 when we adjusted for them. Older participants (≥ 60 years) scored significantly lower than participants aged 18–39 years (p = 0.005) and those aged 40–59 years (p<0.001). Participants with a secondary (p<0.001) or post-secondary (p<0.001) education scored higher than those with only a primary education, and those with no education scored significantly worse that those with any education (p<0.001). Participants who were ethnically Sambaa scored the lowest among the tribal groups which likely reflects their relative lack of education compared to the Chagga tribe which were significantly more likely (p = 0.05) to have a secondary or post-secondary education.

**Table 4:** Mean score for knowledge domain by known-groups.

**Known Groups**	**Mean Score (standard deviation)**	**p-value**
**Gender**		
Male*	3.93 (1.86)	<0.001
Female	3.30 (1.91)	
**Setting**		
Rural	3.06 (1.89)	
Urban*	3.58 (1.91)	0.002
**Age**		
18–39 years old	3.54 (1.84)	0.005
40–59 years old	3.70 (1.91)	<0.001
60+ years old*	2.90 (1.95)	
**Ethnicity**		
Chagga*	3.45 (1.91)	
Pare	3.57 (1.88)	0.72
Sambaa	2.82 (1.88)	0.03
Other	3.61 (1.95)	0.54
**Education**		
None	1.72 (1.51)	<0.001
Primary*	3.33 (1.85)	
Secondary	4.18 (1.84)	<0.001
Post-Secondary	4.74 (1.73)	<0.001
**Occupation**		
Unemployed	3.80 (1.53)	0.05
Farmer/Wage Earner*	3.32 (1.88)	
Small Business/Vendors	3.35 (1.83)	0.97
Professional	3.98 (2.20)	0.06

*Reference group for p-value

## Discussion

We have developed a reliable and valid cross-cultural survey instrument designed to test knowledge, attitudes and practices of chronic kidney disease in a Swahili-speaking population of Northern Tanzania. The final version of the survey consisted of a total of 25 items divided into these three conceptual domains (**S1 Appendix**). We demonstrated content validity through the development of the survey tool (Stage 1), and we demonstrated construct validity through qualitative piloting (Stage 2) and observing the expected differences among the known sub-groups of survey respondents (Stages 3 and 4). Each survey item demonstrated excellent test-retest reliability. We believe that this instrument may be valuable for addressing gaps in NCD care by understanding patient preferences regarding healthcare, formulating educational initiatives and directing development of chronic disease management programs across sub-Saharan Africa.

Although much emphasis is often placed on heuristic analyses for assessing construct validity, the strength of our work is that we have demonstrated construct validity through triangulation of multiple data sources. We piloted the survey through cognitive interviews (Stage 2) in order to establish that each item reflected the underlying referential meanings, and many of the items were derived directly from emerging themes discovered in the qualitative sessions which ensured content validity. We were then able to use the qualitative data to predict the expected performance of the structured instrument administration (Stage 3).

The biggest potential limitation of the known-groups method for evaluating construct validity in cross-cultural surveys are the etic-derived expectations about how certain groups should respond to the items in the instrument. Therefore, we developed our expectations about group convergence and divergence by performing a thematic analysis of the qualitative sessions (Stage 2). This ensured that our expectations were grounded in the data (*a posteriori*) rather than based on pre-formulated assumptions (*a priori*).

Care must be taken when trying to apply this instrument to different settings and populations. Further exploratory work is needed to determine the extent to which the survey remains reliable and valid in other Swahili-speaking populations; however, the transparent methods that we have may be readily applicable to other study areas. Additionally, this thoroughly tested instrument provides a reference for examining the convergent validity of newly developed surveys testing similar constructs.

In conclusion, we used a mixed-methods approach to develop and then validate our cross-cultural survey instrument designed to test knowledge, attitudes and practices of chronic kidney disease in a Swahili-speaking population of northern Tanzania. We believe that these methods and this instrument may be valuable in guiding those conducting related research that seeks to address the growing burden of NCDs.

## Supporting Information

S1 Appendix KAP Survey Instruments (English and Swahili). (DOCX)

S2 Appendix Standard Operating Protocol (SOP) for Household Selection. (DOCX)
