# Supplementary material for: Development and Validation of a Cross-Cultural Knowledge, Attitudes, and Practices Survey Instrument for Chronic Kidney Disease in a Swahili-Speaking Population
Source: PLoS One. 2015 Mar 26;10(3):e0121722. doi: 10.1371/journal.pone.0121722 (PMC4374886; doi:10.1371/journal.pone.0121722)
Supplement: S1 Appendix — (DOCX) [file pone.0121722.s001.docx]

**Development and Validation of a Cross-Cultural Knowledge, Attitudes, and Practices Survey for Chronic Kidney Disease in a Swahili-Speaking Population**

**Supplementary Appendix S1:**

**KAP Survey Instruments (English and Swahili)**

John W Stanifer, MD, MSc^a,b^; Francis Karia, MBA, MSc^c^; Corrine I Voils, PhD^a,d^; Elizabeth L. Turner, PhD^b,e^; Venance Maro, MD, MMed^c^; Dionis Shimbi, BS^c^; Humphrey Kilawe, BS^c^; Matayo Lazaro, BS^c^; Uptal D Patel, MD^a,d, f^

For the

Comprehensive Kidney Disease Assessment for

Risk factors, epIdemiology, Knowledge, and Attitudes (CKD AFRIKA) Study

a Department of Medicine, Duke University; Durham, NC United States

b Duke Global Health Institute, Duke University; Durham, NC United States

c Kilimanjaro Christian Medical College; Moshi, Tanzania

d Health Services Research and Development, Durham Veterans Affairs Medical Center; Durham, NC

e Department of Biostatistics and Bioinformatics, Duke University; Durham, NC United States

f Duke Clinical Research Institute, Duke University; Durham, NC United States

**Survey objective:** To determine knowledge, attitudes, and practices associated with kidney disease.

Date: ___ / ___ / ___

*Information to read to respondent:*

We wish to learn about your knowledge, attitudes and practices regarding kidney disease. We hope to understand your needs and the best way to bring information to you, as well as barriers to seeking medical care. Your individual answers will not be released to anyone and will remain anonymous. Your participation is voluntary and you may choose to stop the interview at any time. This survey should take no longer than 30 minutes. Thank you for your assistance.

The questionnaire was given **orally** ________ or **written** ___________ to the respondent.

**Interviewer:** Place an X in the box of the selected answer(s).

**Knowledge of Kidney Disease: (answer yes, no, do not know, or unsure)**

1. Do you think high blood pressure can cause kidney disease?

| Yes |  |  | No |  |  | Do Not Know |  |  | Unsure |  |  |
| --- | --- | --- | --- | --- | --- | --- | --- | --- | --- | --- | --- |

1. Do you think that high blood sugar (diabetes mellitus) can cause kidney disease?

| Yes |  |  | No |  |  | Do Not Know |  |  | Unsure |  |  |
| --- | --- | --- | --- | --- | --- | --- | --- | --- | --- | --- | --- |

1. Drinking alcohol can cause kidney disease?

| Yes |  |  | No |  |  | Do Not Know |  |  | Unsure |  |  |
| --- | --- | --- | --- | --- | --- | --- | --- | --- | --- | --- | --- |

1. A person can tell if he/she has kidney disease just by the color, quality, or smell of his/her urine?

| Yes |  |  | No |  |  | Do Not Know |  |  | Unsure |  |  |
| --- | --- | --- | --- | --- | --- | --- | --- | --- | --- | --- | --- |

1. Kidney disease can only be diagnosed by a test at the hospital?

| Yes |  |  | No |  |  | Do Not Know |  |  | Unsure |  |  |
| --- | --- | --- | --- | --- | --- | --- | --- | --- | --- | --- | --- |

1. Kidney disease can be prevented if you follow the advice of a Medical Doctor?

| Yes |  |  | No |  |  | Do Not Know |  |  | Unsure |  |  |
| --- | --- | --- | --- | --- | --- | --- | --- | --- | --- | --- | --- |

1. Do the kidneys control body temperature?

| Yes |  |  | No |  |  | Do Not Know |  |  | Unsure |  |  |
| --- | --- | --- | --- | --- | --- | --- | --- | --- | --- | --- | --- |

1. The kidneys filter waste products from the blood?

| Yes |  |  | No |  |  | Do Not Know |  |  | Unsure |  |  |
| --- | --- | --- | --- | --- | --- | --- | --- | --- | --- | --- | --- |

1. Dialysis is a form of treatment for kidney disease?

| Yes |  |  | No |  |  | Do Not Know |  |  | Unsure |  |  |
| --- | --- | --- | --- | --- | --- | --- | --- | --- | --- | --- | --- |

1. Antibiotics are a type of medicine that are used to treat certain infections such as tuberculosis and urinary tract infections (UTIs) among other things.

Antibiotics are a form of treatment for kidney disease?

| Yes |  |  | No |  |  | Do Not Know |  |  | Unsure |  |  |
| --- | --- | --- | --- | --- | --- | --- | --- | --- | --- | --- | --- |

**Attitudes about Kidney Disease: (answer yes or no)**

1. Have you thought that you may have kidney problems?

|  | Yes |  |  | No |  |  |  |
| --- | --- | --- | --- | --- | --- | --- | --- |

1. Do you like the idea of learning all that you can about kidney problems?

|  | Yes |  |  | No |  |  |  |
| --- | --- | --- | --- | --- | --- | --- | --- |

1. If you found out that you have kidney problems, would you be worried about your future?

|  | Yes |  |  | No |  |  |  |
| --- | --- | --- | --- | --- | --- | --- | --- |

1. Would you be worried about your reputation in the community if you found out that you have kidney disease?

|  | Yes |  |  | No |  |  |  |
| --- | --- | --- | --- | --- | --- | --- | --- |

1. Would you be worried about your ability to work if you found out that you have kidney problems?

|  | Yes |  |  | No |  |  |  |
| --- | --- | --- | --- | --- | --- | --- | --- |

1. Would you be worried about your chances of survival if you found out that you have kidney problems?

|  | Yes |  |  | No |  |  |  |
| --- | --- | --- | --- | --- | --- | --- | --- |

1. Do you think that kidney disease is a problem in Kilimanjaro?

|  | Yes |  |  | No |  |  |  |
| --- | --- | --- | --- | --- | --- | --- | --- |

1. Do you think that the cost of kidney disease would be a problem for you?

|  | Yes |  |  | No |  |  |  |
| --- | --- | --- | --- | --- | --- | --- | --- |

**Practices of Kidney Disease: (answer very unlikely, unlikely, likely, or very likely)**

1. If you found out that you have kidney problems…
2. How likely would you be to seek care from a traditional healer?

| Very Unlikely |  | Unlikely |  | Likely |  | Very Likely |  |
| --- | --- | --- | --- | --- | --- | --- | --- |

1. How likely would you be to seek self-treatment at home?

| Very Unlikely |  | Unlikely |  | Likely |  | Very Likely |  |
| --- | --- | --- | --- | --- | --- | --- | --- |

1. How likely would you be to seek care at a hospital or health clinic?

| Very Unlikely |  | Unlikely |  | Likely |  | Very Likely |  |
| --- | --- | --- | --- | --- | --- | --- | --- |

1. Would you be willing to be contacted by cell phone regarding care of your kidneys?

| Very Unlikely |  | Unlikely |  | Likely |  | Very Likely |  |
| --- | --- | --- | --- | --- | --- | --- | --- |

1. Would you be willing to be contacted by email regarding care of your kidneys?

| Very Unlikely |  | Unlikely |  | Likely |  | Very Likely |  |
| --- | --- | --- | --- | --- | --- | --- | --- |

1. Herbal or natural medications are commonly used to treat health problems. Herbal or natural medications may include herbs, teas, foods, creams, lotions, potions, and soups that are used to treat health problems.

How likely would you be to use herbal or natural medications if you found out that you have kidney disease?

| Very Unlikely |  | Unlikely |  | Likely |  | Very Likely |  |
| --- | --- | --- | --- | --- | --- | --- | --- |

1. How likely would you be willing to see a Medical Doctor if you found out that you have kidney problems?

| Very Unlikely |  | Unlikely |  | Likely |  | Very Likely |  |
| --- | --- | --- | --- | --- | --- | --- | --- |

**Malengo ya Uchunguzi:** Kutafuta uelewa, mitazamo, na vitendo kwa ugonjwa wa figo katika jamii kwa ujumla.

Tarehe: ___ / ___ / ___

*Taarifa ya kuwasomea washirki:*

Tunapenda kujifunza kuhusu uelewa, mitazamo,na matendo kuhusiana na ugonjwa wa figo. Tunatumaini kuelewa mahitaji yenu na namna nzuri ya kuleta taarifa kwenu vile vile vikwazo katika utafutaji wa huduma za afya. Majibu yako binafsi hataachiwa mtu yeyote na yatabakia bila majina. Kushiriki kwako ni hiari na unaweza kuchagua kuacha usaili wakati wowote.Uchunguzi huu hautachukua zaidi ya dakika 30. Asante kwa msaada.

Maswali yalitolewa kwa **mdomo** ________ au **maandishi** ___________ kwa mtu anayejibu maswali.

**Anayehoji:** Weka X katika kiboma/sanduku cha majibu yaliyochaguliwa.

**Uelewa wa ugonjwa wa figo: (majibu ndiyo, hapana, sijui, au sina uhakika)**

1. Unafikiri shinikizo la damu linaweza kusababisha ugonjwa wa figo?

| Ndiyo |  | Hapana |  | Sijui |  | Sina uhakika |  |
| --- | --- | --- | --- | --- | --- | --- | --- |

1. Unafikiri sukari nyingi kwenye damu (kisukari) inaweza kusababisha ugonjwa wa figo?

| Ndiyo |  | Hapana |  | Sijui |  | Sina uhakika |  |
| --- | --- | --- | --- | --- | --- | --- | --- |

1. Unywaji wa pombe kupita kiasi unaweza kusababisha ugonjwa wa figo?

| Ndiyo |  | Hapana |  | Sijui |  | Sina uhakika |  |
| --- | --- | --- | --- | --- | --- | --- | --- |

1. Mtu anaweza kusema kama ana ugonjwa wa figo kwa kuangalia rangi, ubora,au harufu ya mkojo wake?

| Ndiyo |  | Hapana |  | Sijui |  | Sina uhakika |  |
| --- | --- | --- | --- | --- | --- | --- | --- |

1. Ugonjwa wa figo unaweza kupimwa kwa vipimo vilivyoko hospitali peke yake?

| Ndiyo |  | Hapana |  | Sijui |  | Sina uhakika |  |
| --- | --- | --- | --- | --- | --- | --- | --- |

1. Ugonjwa wa figo unaweza kuzuilika kwa kufuata ushauri wa daktari?

| Ndiyo |  | Hapana |  | Sijui |  | Sina uhakika |  |
| --- | --- | --- | --- | --- | --- | --- | --- |

1. Figo huwa zina ratibu joto la mwili?

| Ndiyo |  | Hapana |  | Sijui |  | Sina uhakika |  |
| --- | --- | --- | --- | --- | --- | --- | --- |

1. Figo huchuja uchafu kutoka kwenye damu?

| Ndiyo |  | Hapana |  | Sijui |  | Sina uhakika |  |
| --- | --- | --- | --- | --- | --- | --- | --- |

1. Usafishaji wa damu kwa mashine ni mfumo wa kutibu ugonjwa wa figo?

| Ndiyo |  | Hapana |  | Sijui |  | Sina uhakika |  |
| --- | --- | --- | --- | --- | --- | --- | --- |

1. Kiuvijasumu ni aina ya dawa inayotumika kutibu maambukizi mbalimabali kama vile kifua kikuu (TB) na Maambukizi katika njia ya mkojo (UTIs)

Kiuavijasumu (antibiotics) ni aina ya tiba ya kutibu figo?

| Ndiyo |  | Hapana |  | Sijui |  | Sina uhakika |  |
| --- | --- | --- | --- | --- | --- | --- | --- |

**Mtazamo kuhusu ugonjwa wa figo: (jibu ndiyo au hapana)**

1. Umesha wahi kufikiri kuwa una ugonjwa wa figo?

|  | Ndiyo |  |  | Hapana |  |  |  |
| --- | --- | --- | --- | --- | --- | --- | --- |

1. Unapenda wazo la kujifunza kadiri uwezavyo kuhusu ugonjwa wa figo?

|  | Ndiyo |  |  | Hapana |  |  |  |
| --- | --- | --- | --- | --- | --- | --- | --- |

1. Ikiwa utagundua kuwa una matatizo ya figo, utakuwa na wasiwasi kuhusu wakati ujao?

|  | Ndiyo |  |  | Hapana |  |  |  |
| --- | --- | --- | --- | --- | --- | --- | --- |

1. Utakuwa na wasiwasi kuhusiana na mtazamo wa jamii juu yako iwapo utagundua kuwa una ugonjwa wa figo?

|  | Ndiyo |  |  | Hapana |  |  |  |
| --- | --- | --- | --- | --- | --- | --- | --- |

1. Utakuwa na wasiwasi kuhusu uwezo wako wa kufanya kazi iwapo utagundua kuwa una ugonjwa wa figo?

|  | Ndiyo |  |  | Hapana |  |  |  |
| --- | --- | --- | --- | --- | --- | --- | --- |

1. Utakuwa na hofu ya uwezekano wa kupona iwapo utagundua una matatizo ya figo?

|  | Ndiyo |  |  | Hapana |  |  |  |
| --- | --- | --- | --- | --- | --- | --- | --- |

1. Unafikili ugonjwa wa figo ni tatizo Kilimanjaro?

|  | Ndiyo |  |  | Hapana |  |  |  |
| --- | --- | --- | --- | --- | --- | --- | --- |

1. Unafikiri gharama za ugonjwa wa figo inaweza kuwa tatizo kwako?

|  | Ndiyo |  |  | Hapana |  |  |  |
| --- | --- | --- | --- | --- | --- | --- | --- |

**Matendo kufuatia Ugonjwa wa figo: (jibu haiwezekani kabisa, haiwezekani, inawezekana, au inawezekana kabisa)**

1. Iwapo utagundua kuwa una matatizo ya figo…
2. Kuna uwezekano gani wa wewe kutafuta dawa kwa mganga wa kienyeji?

­­­­­­­­­­­­­­

| Haiwezekani kabisa |  | Haiwezekani |  | Inawezekana |  | Inawezekana kabisa |  |
| --- | --- | --- | --- | --- | --- | --- | --- |

1. Kuna uwezekano gani wa wewe kutafuta matibabu binafsi nyumbani?

| Haiwezekani kabisa |  | Haiwezekani |  | Inawezekana |  | Inawezekana kabisa |  |
| --- | --- | --- | --- | --- | --- | --- | --- |

1. Kuna uwezekano gani wa wewe kutafuta uangalizi/tiba kwenye hospitali au vituo vya afya?

| Haiwezekani kabisa |  | Haiwezekani |  | Inawezekana |  | Inawezekana kabisa |  |
| --- | --- | --- | --- | --- | --- | --- | --- |

1. Utaridhia kupata mawasiliano kupitia simu yako ya mkononi kuhusiana na matibabu ya figo zako?.

| Haiwezekani kabisa |  | Haiwezekani |  | Inawezekana |  | Inawezekana kabisa |  |
| --- | --- | --- | --- | --- | --- | --- | --- |

1. Utaridhia kupata mawasiliano kwa njia ya barua pepe kuhusiana na matibabu yako ya figo?

| Haiwezekani kabisa |  | Haiwezekani |  | Inawezekana |  | Inawezekana kabisa |  |
| --- | --- | --- | --- | --- | --- | --- | --- |

1. Mitishamba na dawa za asili ni huwa kwa kawaida vinatumika kutibu matatizo ya kiafya. Mitishamba na dawa za asili ni pamoja na mimea, chai, chakula, krimu, losheni, dawa inayosemekana kuwa na athari za kichawi na supu vinatumika kutibu matatizo ya kiafya.

Kuna uwezekano gani wa wewe kutumia mitishamba na dawa za asili ikiwa utagundua kuwa una ugonjwa wa figo?

| Haiwezekani kabisa |  | Haiwezekani |  | Inawezekana |  | Inawezekana kabisa |  |
| --- | --- | --- | --- | --- | --- | --- | --- |

1. Kuna uwezekano kiasi gani kwako kwenda kwa hiari kumuona daktari iwapo utapatikana kuwa na matatizo ya figo?

| Haiwezekani kabisa |  | Haiwezekani |  | Inawezekana |  | Inawezekana kabisa |  |
| --- | --- | --- | --- | --- | --- | --- | --- |
