# Supplementary material for: Development and Validation of a Cross-Cultural Knowledge, Attitudes, and Practices Survey Instrument for Chronic Kidney Disease in a Swahili-Speaking Population
Source: PLoS One. 2015 Mar 26;10(3):e0121722. doi: 10.1371/journal.pone.0121722 (PMC4374886; doi:10.1371/journal.pone.0121722)
Supplement: S2 Appendix — (DOCX) [file pone.0121722.s002.docx]

**Development and Validation of a Cross-Cultural Knowledge, Attitudes, and Practices Survey for Chronic Kidney Disease in a Swahili-Speaking Population**

**Supplementary Appendix S2:**

**Standard Operating Protocol (SOP) for Household Selection**

John W Stanifer, MD, MSc^a,b^; Francis Karia, MBA, MSc^c^; Corrine I Voils, PhD^a,d^; Elizabeth L. Turner, PhD^b,e^; Venance Maro, MD, MMed^c^; Dionis Shimbi, BS^c^; Humphrey Kilawe, BS^c^; Matayo Lazaro, BS^c^; Uptal D Patel, MD^a,d, f^

For the

Comprehensive Kidney Disease Assessment for

Risk factors, epIdemiology, Knowledge, and Attitudes (CKD AFRIKA) Study

a Department of Medicine, Duke University; Durham, NC United States

b Duke Global Health Institute, Duke University; Durham, NC United States

c Kilimanjaro Christian Medical College; Moshi, Tanzania

d Health Services Research and Development, Durham Veterans Affairs Medical Center; Durham, NC

e Department of Biostatistics and Bioinformatics, Duke University; Durham, NC United States

f Duke Clinical Research Institute, Duke University; Durham, NC United States

PURPOSE:

To provide a reproducible, systematic, and random method of selecting households for sampling.

DEFINITIONS:

- Cluster = randomly, pre-selected geographic location that includes multiple households for sampling
- Dwelling= A free-standing building that is covered by a roof. Buildings that share a foundation or appear to share a foundation should be considered as one dwelling.
- Household = Persons residing within a dwelling whose food is prepared by the same person(s)
- ID = Unique Identification Number that is assigned to each participant and each household.
- Household ID= Two digit Unique Identification Number contained in the study ID number that is assigned to each household.
- Eligible Individuals: Adults over the age of 18 who are not pregnant. Ex-pats or Temporary Residents should be excluded unless they are FULL citizens who reside in Kilimanjaro, Tanzania full time (i.e. more than 9 months out of every year).

OVERVIEW:

- Cluster site identification
- Household identification
- Household selection process

PROCESS:

1. Cluster site identification: the starting point from which household selection will occur has been identified based on a random GPS coordinates.
2. The dwelling physically closest to the starting point will be approached first.
3. Household Selection Process
   1. The first dwelling should be approached:
      1. If that dwelling fulfills the definition of a household then assign it a household ID and assess the eligibility of the household adults according to the enrollment protocol.
      2. If that dwelling does NOT fulfill the definition of a household then move on to the next dwelling.
      3. Unless the dwelling is clearly marked as a business, shop, or restaurant then the field surveyors should assume that it could be a household. They should then approach to confirm. (*Remember that sometimes people who own shops also live in the back – if any doubt then they should always approach to confirm*).
   2. To identify the next dwelling to approach for sampling, the following methods should be used:
      1. The field surveyor will stand with his/her back to the main entrance of the first dwelling.
      2. Flip a coin.
      3. If the coin lands on TAILS then proceed to your LEFT. If the coin lands on HEADS then proceed to on your RIGHT.
      4. Next, roll the die to determine which house to approach. The numbers on the die represent which house number (in sequential order according to physical distance to the front door) will be chosen.
      5. If the surveyor comes to an intersection or dead-end before reaching the house number on the die, then flip the coin again to determine the continuing direction. Again, TAILS will be LEFT and HEADS will be RIGHT.
      6. In instances where there is only one physical direction to go, then proceed in that direction.
      7. If a dwelling repeats, then repeat the coin-flip and die process.
4. Protocol for Gated Houses
   - - - 1. House with a Gatekeeper

First, contact the gatekeeper to explain our intentions. If agreeable, he may allow entry.

If not agreeable to entry, then leave a study overview pamphlet along with our contact information.

Arrange a follow-up time to see if the owners have expressed interest.

- - - - 1. Closed Gate House

If a gatekeeper is present then proceed as above.

If no gatekeeper and no way to contact the household members, then record as non-response.

Two additional visits, including one off-hours visit (i.e. evening or weekend day), should be attempted according to the follow-up protocol.

- - - - 1. Open Gate

First, ensure that there is no gatekeeper.

If no gatekeeper, then approach the household as you would any other dwelling.
